# Involvement of EupR, a response regulator of the NarL/FixJ family, in the control of the uptake of the compatible solutes ectoines by the halophilic bacterium *Chromohalobacter salexigens*

**DOI:** 10.1186/1471-2180-10-256

**Published:** 2010-10-13

**Authors:** Javier Rodríguez-Moya, Montserrat Argandoña, Mercedes Reina-Bueno, Joaquín J Nieto, Fernando Iglesias-Guerra, Mohamed Jebbar, Carmen Vargas

**Affiliations:** 1 Department of Microbiology and Parasitology, Faculty of Pharmacy, University of Seville, Seville, Spain; 2 Department of Organic and Pharmaceutical Chemistry. Faculty of Pharmacy, University of Seville, Seville, Spain; 3 Laboratoire Interactions Cellulaires et Moléculaires, DUALS, CNRS 6026, Université de Rennes I, Campus Beaulieu, 35042 Rennes Cedex, France; 4 Laboratoire de Microbiologie des Environnements Extrêmes - LM2E (CNRS), Université de Bretagne Occidentale, Plouzané, France

## Abstract

**Background:**

Osmosensing and associated signal transduction pathways have not yet been described in obligately halophilic bacteria. *Chromohalobacter salexigens *is a halophilic bacterium with a broad range of salt tolerance. In response to osmotic stress, it synthesizes and accumulates large amounts of the compatible solutes ectoine and hydroxyectoine. In a previous work, we showed that ectoines can be also accumulated upon transport from the external medium, and that they can be used as carbon sources at optimal, but not at low salinity. This was related to an insufficient ectoine(s) transport under these conditions.

**Results:**

A *C. salexigens *Tn*1732*-induced mutant (CHR95) showed a delayed growth with glucose at low and optimal salinities, could not grow at high salinity, and was able to use ectoines as carbon sources at low salinity. CHR95 was affected in the transport and/or metabolism of glucose, and showed a deregulated ectoine uptake at any salinity, but it was not affected in ectoine metabolism. Transposon insertion in CHR95 caused deletion of three genes, Csal0865-Csal0867: *acs*, encoding an acetyl-CoA synthase, *mntR*, encoding a transcriptional regulator of the DtxR/MntR family, and *eupR*, encoding a putative two-component response regulator with a LuxR_C-like DNA-binding helix-turn-helix domain. A single *mntR *mutant was sensitive to manganese, suggesting that *mntR *encodes a manganese-dependent transcriptional regulator. Deletion of *eupR *led to salt-sensitivity and enabled the mutant strain to use ectoines as carbon source at low salinity. Domain analysis included EupR as a member of the NarL/FixJ family of two component response regulators. Finally, the protein encoded by *Csal869*, located three genes downstream of *eupR *was suggested to be the cognate histidine kinase of EupR. This protein was predicted to be a hybrid histidine kinase with one transmembrane and one cytoplasmic sensor domain.

**Conclusions:**

This work represents the first example of the involvement of a two-component response regulator in the osmoadaptation of a true halophilic bacterium. Our results pave the way to the elucidation of the signal transduction pathway involved in the control of ectoine transport in *C. salexigens*.

## Background

Due to the frequent osmolarity changes in their habitat, microorganisms have developed a number of osmoadaptation mechanisms to adapt to these fluctuations [1,2]. In most bacteria, the long-term response to hyperosmotic conditions involves the intracellular accumulation of large quantities of small, specific organic osmolytes called compatible solutes since they do not interfere with the normal functioning of the cell [3]. It has been demonstrated that compatible solutes have the ability to protect enzymes and whole cells against different stresses such as those caused by salt, heating, freezing and desiccation [3,4]. Thus, they are considered as biostabilizers. It is commonly accepted that uptake of exogenous compatible solutes (osmoprotectants) is preferred over their synthesis de novo, as it is energetically less costly [5]. On the other hand, hypoosmotic stress leads to opening of mechanosensitive channels, which function as emergence valves leading to rapid efflux of compatible solutes thereby lowering the osmotic driving force for water entry [6]. Besides their role as stress protectants, some compatible solutes can be used as carbon, energy or nitrogen sources. This duality of functions (stress protection and nutrition) requires complex regulatory circuits (most of them not yet elucidated) to adjust the rate of compatible solute biosynthesis, transport and catabolism [4,7,8].

A number of genes and enzymes responsible for synthesis, uptake and efflux of compatible solutes have been identified in diverse bacteria [1,6-10]. However, the mechanisms by which bacteria sense osmotic shifts (osmosensing) and the signal transduction pathways leading to these genes (osmosignaling) have focused on membrane-based osmosensors from moderately halotolerant, but not halophilic, bacteria. These include osmosensory transporters, histidine kinases of two-component transcriptional regulatory systems [9], and mechanosensitive channels of the MscL, MscS and MscK type [6]. Whereas the first and the third group can detect osmotic pressure changes and respond by mediating compatible solute uptake or efflux, respectively, without the assistance of other proteins, membrane-bound histidine kinases detect changes in osmotic pressure and other signals and then respond by directing cognate response regulators to modulate transcription of osmoregulated genes. The best studied osmosensory transporters mediate uptake of potassium, i.e. Trk from *Escherichia coli*, and betaine, such as ProP from *E. coli*, OpuA from *Lactococcus lactis *and BetP from *Corynebacterium glutamicum *[9,11]. On the other hand, the best characterized two-component transcriptional regulatory systems involved in bacterial osmoadaptation are KdpDE and EnvZ/OmpR from *E. coli*, and MtrAB from *C. glutamicum *[11-13].

Both sensory histidine protein kinases and response regulators of two-component signal transduction systems are multi-domain proteins. Histidine protein kinases typically consist of a variable N-terminal sensory or "input" domain, which detects environmental stimuli and activates a conserved C-terminal cytoplasmic transmitter domain, comprising an ATP-binding kinase domain and a histidine-containing dimerization domain. On the other hand, most response regulators contain a conserved N-terminal receiver (REC) domain and a variable C-terminal effector or "output" domain. The first one catalyzes the transfer of the phosphoryl group from the cognate histidine protein kinase to one of its own aspartic residues. As a result, the receiver domain undergoes a conformational change capable of promoting activity of the effector domain [14,16].

Two general approaches have been used for classifying bacterial two-component systems. The first one is based on the diversity of input (i.e. cellular location, membrane topology, arrangement of sensory domains) or output (i.e., DNA-binding, RNA-binding, protein-binding, enzymatic, etc) domain architecture and domain combinations [14,15,17]. The second one is based on the phylogeny of transmitter and receiver domains [18]. Interestingly, the results of both classifications agree to a certain extent, as it seems that the majority of signal transduction proteins belong to a relatively small number of major families, which share common ancestry, and gene/domain architecture. Osmosensing and associated signal transduction pathways have not yet been described in obligate halophilic bacteria. *Chromohalobacter salexigens *[19] is a halophilic gamma proteobacterium that grows optimally at 1.5 M NaCl in minimal medium [20]. It requires at least 0.5 M NaCl for any growth at all, and can tolerate up to 3 M NaCl, being considered as a model microorganism to study prokaryotic osmoadaptation [8]. Interestingly, *C. salexigens *lowest salinity for growth is the highest NaCl concentration that the non halophilic *E. coli*, traditionally used for osmoregulation studies, can tolerate. *C. salexigens *finely adjusts its cytoplasmic compatible solute pool in order to cope with high salinity and supra-optimal temperatures [21,22]. This is achieved by a highly hierarchical accumulation of solutes, dominated by the uptake of external osmoprotectants such as betaine or its precursor choline [23,24], and followed by the synthesis of endogenous solutes, mainly ectoines (ectoine and hydroxyectoine), and minor amounts of glutamate, glutamine, trehalose and glucosylglycerate [8]. Ectoine and hydroxyectoine are essential for osmoprotection and thermoprotection, respectively [22].

*C. salexigens *can also accumulate ectoines after transport from the external medium, and the ectoine transport rate is maximal at optimal salinity [25]. Within the sequence of the *C. salexigens *genome, we have found orthologs to the TRAP-T-type TeaABC transport system for ectoines of the closely related *Halomonas elongata *[10]. We have experimental evidence that this system is the main responsible for the uptake of ectoines in *C. salexigens *(J. Rodriguez-Moya, unpublished data). On the other hand, although glucose is the preferred carbon and energy source, *C. salexigens *can use a wide range of substrates as nutrients, including the compatible solutes betaine, ectoine and hydroxyectoine [25]. Remarkably, neither ectoines nor betaine could support *C. salexigens *growth at low salinity, most probably due to an insufficient uptake of these compatible solutes [25].

Osmoadaptive response through ectoine(s) synthesis in *C. salexigens *seems to be finely controlled at the transcriptional level, and several general (σ^S^, σ^32^, Fur) or specific regulators have been described [8,24]. However, the associated sensors remain to be elucidated. In addition, information on osmosensing and signal transduction pathways leading to osmoprotectant uptake in *C. salexigens *is missing. In this work, we isolated a *C. salexigens *salt-sensitive mutant, strain CHR95, which was nevertheless able to use ectoines as a sole carbon source at low salinities due to a deregulated transport. This mutant was affected in three genes, two of which were transcriptional regulators. Analyses of single mutants affected in these regulators suggested the protein EupR as the response regulator of a two-component system involved in the regulation of ectoine(s) uptake. In addition, we predicted and analyzed its putative sensor histidine kinase. This work establishes the first analysis of the involvement of the response regulator of a two-component system in the osmoadaptive response of halophilic bacteria.

## Results

### *C. salexigens *mutant CHR95 can use ectoines as the sole carbon sources at low salinity

**Figure 1:**
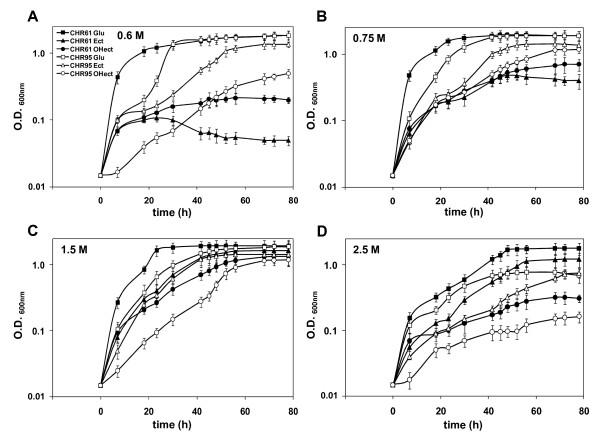
***C. salexigens *CHR95 can use ectoine as the sole carbon source at low salinity**. Wild type (solid symbols) and CHR95 (open symbols) strains were grown at 37°C in M63 minimal medium with 20 mM glucose, 20 mM ectoine, or 20 mM hydroxyectoine and 0.6 (A), 0.75 (B), 1.5 (C) and 2.5 (D) M NaCl. Values shown are the mean of two replicas of each conditions in three independent experiment ± SD (standard deviation)

*C. salexigens *is able to grow in M63 minimal medium with 0.5 to 3 M NaCl. In a search for *C. salexigens *salt-sensitive mutants, strain CHR95 was isolated after Tn*1732 *transponson mutagenesis, as being able to grow at 0.5 M but not at 2.7 M NaCl on M63 plates (see Methods). To further characterize its salinity range, *C. salexigens *wild type and CHR95 strains were grown in M63 minimal medium with 20 mM glucose as the sole carbon source, at salinities ranging from 0.6 to 2.5 M NaCl. As shown in Figure 1, at 0.6 M NaCl the growth curve of strain CHR95 showed a 20 h lag phase, followed by a sharp exponential phase to reach the same OD_600 _as the wild type strain after ca. 30 h of growth (see Table 1 for growth rates). At 0.75 M and 1.5 M NaCl, growth of the mutant followed a similar pattern, i.e., an extended lag phase, followed by a less pronounced exponential phase than that of the wild type strain, to eventually reach the wild type growth curve at the stationary phase of growth. At 2.5 M NaCl the strain CHR95 showed a salt-sensitive phenotype, as its growth curve did not reach an OD_600 _above 0.6 units (Figure 1 and Table 1).

**Table 1:** Growth rates of *C. salexigens *wild type strain (CHR61) and mutant CHR95 on glucose and ectoines at different salinities

Strain and carbon source	**Growth rate (h**^**-1**^**)**
**CHR61 **glucose	
0.6 M	0.043
0.75 M	0.066
1.5 M	0.100
2.5 M	0.061
**CHR61 **ectoine	
0.6 M	0
0.75 M	0.013
1.5 M	0.045
2.5 M	0.032
**CHR61 **hydroxyectoine	
0.6 M	0
0.75 M	0.012
1.5 M	0.030
2.5 M	0.007
**CHR95 **glucose	
0.6 M	0.090
0.75 M	0.055
1.5 M	0.044
2.5 M	0.007
**CHR95 **ectoine	
0.6 M	0.038
0.75 M	0.045
1.5 M	0.046
2.5 M	0.020
**CHR95 **hydroxyectoine	
0.6 M	0.010
0.75 M	0.023
1.5 M	0.045
2.5 M	0

We also compared the ability of the *C. salexigens *wild type strain and mutant CHR95 to use ectoine and hydroxyectoine as the sole carbon sources at different salinities. As shown in Figure 1 and Table 1, in all growth experiments ectoine was better carbon source than hydroxyectoine. Ectoine and hydroxyectoine did not support the growth of the wild type strain at low salinity (0.6 M NaCl), and growth was severely impaired at 0.75 M NaCl). They were used as carbon sources at optimal (1.5 M NaCl) and high (2.5 M NaCl) salinity (in this latter case, only ectoine and after a prolonged lag phase). Remarkably, mutant CHR95 was able to use ectoine and hydroxyectoine as the sole carbon and energy source at low salinities (0.6-0.75 M NaCl), although growth with hydroxyectoine was initiated after a long lag phase (Figure 1 and Table 1). Other compatible solutes like glycine betaine were not metabolized under low salinity conditions (not shown). At 1.5 M NaCl with ectoine or hydroxyectoine, growth of the mutant was delayed, if compared to the wild type strain, whereas at 2.5 M NaCl ectoine or hydroxyectoine did weakly support or not, respectively, CHR95 growth (Figure 1 and Table 1).

Given that strain CHR95 showed a delayed growth with glucose at any salinity tested, we used natural abundance ^13^C-NMR to determine the total pool of compatible solutes accumulated by cells grown in M63 with 2.5 M NaCl. The ^13^C-NMR spectrum of the mutant contained four sets of resonances that were assigned to ectoine, hydroxyectoine, glutamate and glutamine (not shown). This observation suggested that CHR95 was not affected in the genes encoding the synthesis of compatible solutes.

### Mutant CHR95 is affected in the transport and metabolism of glucose

**Figure 2:**
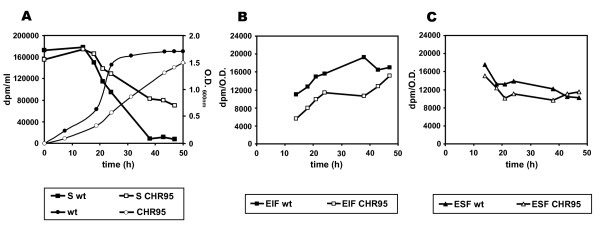
***C. salexigens *CHR95 is affected in the transport and metabolism of glucose**. Cells grown in M63 with 1.5 M NaCl up to exponential phase were centrifuged, resuspended in the same medium to an OD600 of c. 0.6, supplemented with 100 μM of [^14^C]-glucose. After different times of incubation at 37°C, the glucose remaining in the supernatant (S) and cytoplasmatic solutes synthesized from ectoine, present in the ethanol insoluble (EIF) and soluble (ESF) fractions, respectively, were determined as described in Methods. The data are the averages of three different replicates ± SD (standard deviation).

Since, if compared to the wild type strain, strain CHR95 showed delayed growth with glucose at low and optimal salinity, we analyzed the metabolism of glucose in both strains. For this purpose, cells were cultivated in M63 with 1.5 M NaCl, and the fate of radioactive glucose was determined at different time intervals as described in Methods (Figure 2). First, the total radioactivity remaining in supernatant (S) was determined and considered as an indirect measure of glucose transport. As evidenced by the sharp decrease in the radioactivity remaining in the supernatant, the wild type strain incorporated about 95% of the glucose from 20 (early exponential phase) to 38 hours of incubation. In contrast, glucose uptake by the mutant was slower, with 10-fold higher radioactivity levels in its supernatant than those of the wild type after 38 hours of incubation (Figure 2a). Second, we determined, for the wild type and CHR95 strains, the radioactivity present in the ethanol insoluble fraction (EIF), containing cell envelopes and intracellular macromolecules (lipids, proteins), and the ethanol soluble fraction (ESF), containing small cytoplasmic organic solutes (including ectoines, amino acids, and others). From the same time interval comprised between 20 and 38 hours of incubation, the radioactivity present in the EIF and the ESF of strain CHR95 was 1.5 to 1.8-fold lower (Figure 2b), and 1.3-fold lower (Figure 2c), respectively, than those of the wild type strain. These results, taken together, suggest that the slow growth of strain CHR95 with glucose might be due, at least in part, to a decreased glucose transport and metabolism.

### Mutant CHR95 possesses a deregulated ectoine uptake

**Figure 3:**
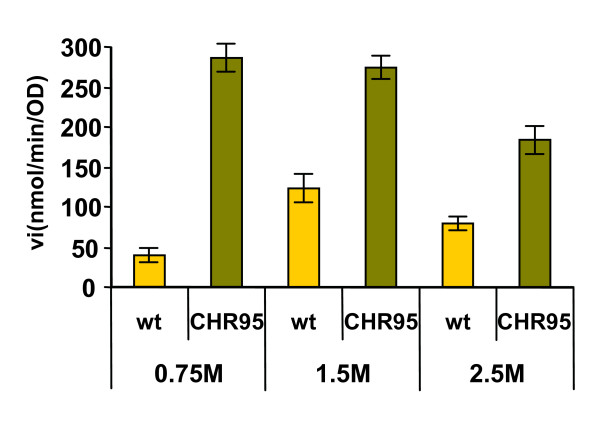
***C. salexigens *CHR95 shows a deregulated ectoine uptake**. The wild-type strain and the mutant CHR95 (Δ*acseupRmntR*::Tn*1732*) were grown in glucose M63 minimal medium containing the indicated concentration of NaCl. The measurement of 40 [^14^C]-ectoine uptake rates (vi, expressed as nmol min^-1 ^OD^-1 ^units) was performed as described in Methods. Experiments were repeated twice, and the data correspond to mean values.

As mutant CHR95, but not the wild type strain, could use ectoines as nutrients at low salinities, we investigated the transport and metabolism of ectoine in both strains in response to increasing osmolarity. As previously reported by Vargas et al [25], the wild type strain showed its maximal ectoine transport rate at the optimal salinity for growth (1.5 M NaCl), which was 3- and 1.5-fold higher than those observed at 0.75 and 2.5 M NaCl, respectively (Figure 3). Notably, the ectoine transport rates of strain CHR95 were 8-, 2.3-, and 2.5-fold higher at 0.75, 1.5, and 2.5 M NaCl, respectively, than those of the wild type grown at the same salt concentrations (Figure 3).

**Figure 4:**
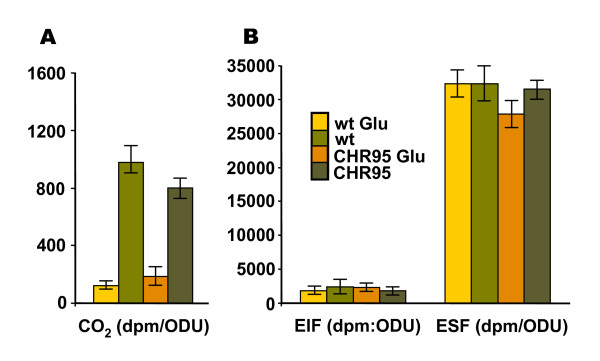
***C. salexigens *CHR95 is not affected in the metabolism of ectoine**. Cells grown in M63 with 1.5 M NaCl up to exponential phase were centrifuged, resuspended in the same medium to an OD_600 _of ca. 0.6, supplemented with 87 μM of [^14^C]-ectoine and incubated with and without 20 mM of glucose. After 2 h incubation at 37°C, CO_2 _production from ectoine (A) and macromolecules (EIF, B) and cytoplasmatic solutes (ESF, C) synthesized from ectoine, present in the ethanol insoluble and soluble fractions, respectively, were determined as described in Methods. The data are the averages of three different replicates ± SD (standard deviation).

To test if the metabolism of ectoine was affected in CHR95, the fate of radioactive ectoine was analysed in the presence or absence of 20 mM glucose as described in Methods, and compared to that of the wild type strain. According to previous studies [25], CO_2 _production due to ectoine catabolism in the wild type strain was lower (40-fold) in the presence of glucose, suggesting that ectoine utilization is partially repressed by glucose. No significant differences were found between CO_2 _production from ectoine by CHR95 and the wild type strain, neither with nor without glucose addition (Figure 4a). In both strains, most of the carbon backbone of ectoine (ca. 70% of the total radioactivity added) was found in the ethanol soluble fraction (ESF), whereas only about 3.82% of the total radioactivity added was found in the ethanol insoluble fraction (EIF). No significative differences were found in the radioactivity present in the ESF and EIF fractions of the wild type and mutant strain. Glucose did not influence the biosynthesis of molecules from ectoine in any of these fractions (Figure 4b). These results suggested that whereas ectoine transport is deregulated in mutant CHR95 at any salinity, ectoine metabolism is not affected in this strain.

### Transposon insertion in mutant CHR95 caused deletion of genes for the acetyl-CoA synthase and two transcriptional regulators

**Figure 5:**
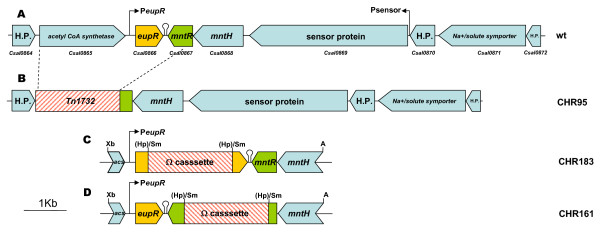
**Genetic organization of the *C. salexigens eupR *region and constructions derived from it**. *(A) C. salexigens *genomic region containing *eupR *and *Csal869*, encoding its putative cognate histidine kinase, the *mntH*-*mntR *genes related to manganese transport, and the *acs *gene encoding a putative acetyl-CoA synthase. Promoters are indicated by angled arrows. The transcriptional terminator downstream of *eupR *is shown as a lollipop. (B) The same genomic region in *C. salexigens *CHR95. The insertion of Tn*1732 *deleted *acs*, *eupR *and *mntR*. (C) Generation of the *eupR *strain. *eupR *was inactivated by the insertion of an Ω*aac *cassette, which carries resistance genes for geneticin and gentamicin, into its unique site *Hpa*I site (H). (D) Generation of the *mntR *strain. *mntR *was inactivated by the insertion of an Ω cassette, which carries resistance genes for streptomycin and spectinomycin, into its unique site *Hpa*I site (H).

The salt sensitivity of strain CHR95, together with its altered glucose metabolism and its capacity to use ectoines as carbon sources at low salinity, prompted us to analyze the gene(s) that was(were) affected by the Tn*1732 *insertion in this mutant. For this purpose, the DNA region flanking the insertion was cloned in plasmid pRR1, which was shown to carry Tn*1732 *(6.7-kb) plus about 14 kb of adjacent DNA. To exactly localize the gene(s) disrupted by the transposon, the DNA region flanking the insertion was sequenced by using Tn*1732 *internal primers. As shown in Figure 5, three genes were deleted by the Tn*1732 *insertion, named as *Csal0865*, *Csal0866*, and *Csal0867 *within the *C. salexigens *genome sequence. *Csal0865*and Csal0866 were located in the forward strand and separated by a 260-bp intergenic region, whereas *Csal0867 *was located in the complementary strand. The product of *Csal0865 *(hereafter Acs) was annotated as an acetyl CoA synthase, which activates acetate to acetyl-CoA. In an iterative PSI-BLAST search, it showed ca 70% of amino acid identity to proteins annotated as acetyl CoA synthases from *Rhodopseudomonas palustris *and *Vibrio cholerae*. Genes *Csal0866 *and *Csal0867 *were predicted to encode putative transcriptional regulators. Thus, the *Csal0866 *product (hereafter EupR) was annotated as a "two-component LuxR family transcriptional regulator". An iterative PSI-BLAST search revealed a high identity (ca. 65-70%) to proteins annotated as response regulators of gamma (i.e. *Vibrio*, *Pseudomonas*, *Shewanella*, *Marinobacter, Aeromonas*) and alpha (ie. *Bradyrhizobium, Labrenzia*) proteobacteria. On the other hand, the protein encoded by *Csal0867 *(hereafter MntR) showed a high identity to manganese-dependent transcriptional regulators of the DtxR/MntR family such as MntR of *E. coli*. Moreover, it showed the characteristic domains of these metalloregulators, i.e., an N-terminal helix-turn-helix domain and a C-terminal metal binding and dimerisation domain. *mntR *was preceded by two genes encoding a putative sensor histidine kinase (Csal869) and a putative manganese transporter (MntH), respectively. An *in silico *analysis of promoter and transcriptional terminator regions showed two putative σ^70^-dependent promoters (one upstream of *eupR *and another one upstream of the sensor histidine kinase-encoding gene), and a putative Rho-independent transcriptional terminator downstream of *eupR*.

### The *C. salexigens *MntR regulator is involved in the control of manganese uptake

**Figure 6:**
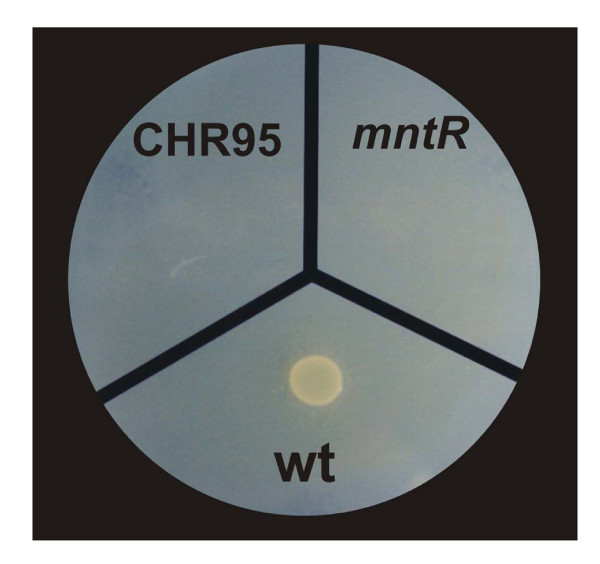
***C. salexigens *MntR is involved in the control of manganese uptake**. 100 μL of overnight cultures of the wild type, CHR95 (Δ*acseupRmntR::Tn1732*) and CHR 161 (*mntR*::Ω) were placed on SW2 plates with 0.5 mM MnCl_2 _and growth was observed after incubation at 37°C for 48 h.

In other bacteria, such as *Bacillus subtilis*, MntR is a manganese-dependent metalloprotein involved in the regulation of manganese uptake. *mntR *mutants are manganese-sensitive since MntR represses genes encoding Mn(II) transporters. Thus, in the absence of MntR, manganese uptake is deregulated and therefore manganese is toxic to the cells [26]. Since the gene *Csal0867 *(encoding a putative MntR/DtxR-like global transcriptional regulator) was deleted by the Tn*1732 *insertion in strain CHR95, we generated a *mntR *strain (CHR161), in which the gene encoding this transcriptional regulator was interrupted by an omega cassette (Figure 5), and investigated its sensitivity to manganese. The wild type, *mntR*, and CHR95 strains were plated on modified SW-2 plates with different MnCl_2 _concentrations ranging from 0.5 to 2.5 mM. As expected, mutants CHR95 and CHR161 (*mntR*) did not grow with any MnCl_2 _concentration (Figure 6). This finding, together with the *in silico *analysis of the motifs in the protein encoded by Csal0867, suggested that the *mntR *gene might encode a manganese-dependent transcriptional regulator.

### Deletion of the *eupR *gene in the CHR95 mutant is responsible for deregulation of ectoine uptake

**Figure 7:**
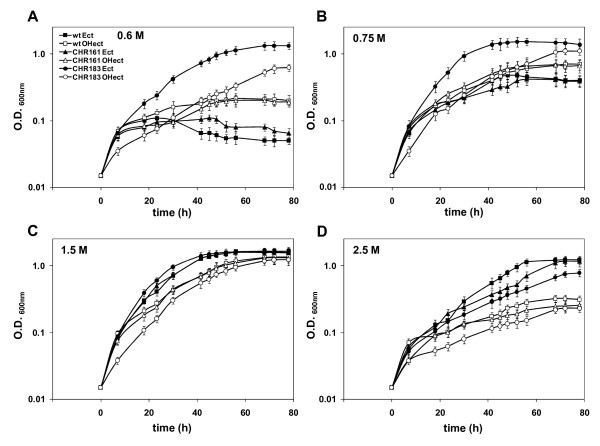
***C. salexigens *EupR is involved in the control of ectoine uptake**. Wild type strain (squares), CHR161 mutant (*mntR*::Ω) (triangles) and CHR183 mutant (*eupR*::Ω*aac*) (circles) were grown at 37°C in M63 medium with 20 mM ectoine (black markers) or 20 mM hydroxyectoine (white markers) and 0.6 (A), 0.75 (B) or 1.5 (C) M NaCl. Values shown are the mean of two replicas of each condition in three independent experiment ± SD (standard deviation)

The results presented so far suggested that at least one of the genes affected by the Tn*1732 *transposon insertion in *C. salexigens *CHR95 could be involved in the regulation of ectoine uptake. Besides the gene encoding the MntR regulator, the gene *Csal0866 *(*eupR*), encoding a response regulator of a two-component system, was deleted by the Tn*1732 *insertion in CHR95 (Figure 5). Thus, we generated a mutant affected in this transcriptional regulator (CHR183) and tested its capacity to use ectoines as carbon source at low salinity, as a measure of enhanced transport at this salinity. The wild type and CHR161 (*mntR*) strains were also included in the assay for comparative purposes. Strains were grown in M63 medium with glucose, ectoine or hydroxyectoine as the sole carbon sources, at salinities ranging from 0.6 to 2.5 M NaCl. No significant differences were found between the growth of the *mntR *mutant and the wild type strain with any carbon source at any salinity tested (Figure 7 and Table 2). In contrast, mutant CHR183 (*Csal0866*) reproduced the phenotype of strain CHR95 and was able to use ectoine and, to a lower extent, hydroxyectoine as the sole carbon and energy sources at low salinity (Figure 7 and Table 2). Like strain CHR95, and if compared to the wild type, growth of CHR183 (*Csal0866*) with glucose was delayed from 0.6 to 1.5 M NaCl, and severely impaired at 2.5 M NaCl (data not shown). The above findings suggest that deletion of gene *Csal0866 *enables the strain to use ectoines as carbon source at low salinity, as a consequence of ectoine transport deregulation at this salinity. Therefore, the product of *Csal0866 *was named EupR (after Ectoine uptake Regulator).

**Table 2:** Growth rates of *C. salexigens *strains CHR161 (*mntR*) and CHR183 (*eupR*) on ectoines at different salinities

Strain and carbon source	**Growth rate (h**^**-1**^**)**
**CHR161 **ectoine	
0.6 M	0
0.75 M	0.011
1.5 M	0.041
2.5 M	0.029
**CHR161 **hydroxyectoine	
0.6 M	0
0.75 M	0.012
1.5 M	0.024
2.5 M	0
**CHR183 **ectoine	
0.6 M	0.033
0.75 M	0.044
1.5 M	0.040
2.5 M	0.016
**CHR183 **hydroxyectoine	
0.6 M	0.015
0.75 M	0.021
1.5 M	0.023
2.5 M	0

### EupR is a response regulator of the NarL/FixJ family of proteins

**Figure 8:**
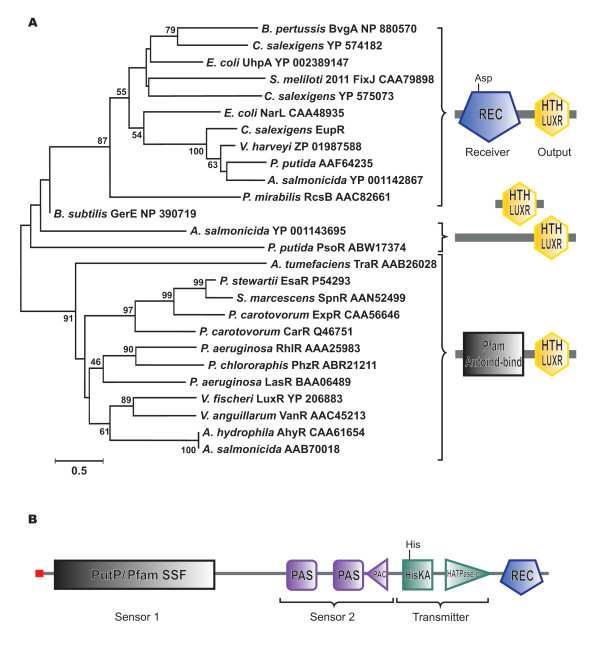
***In silico *analysis of EupR and its putative cognate histidine kinase**. **(A) **EupR is a two-component response regulator of the NarL/FixJ family of proteins. Neighbor-Joining tree based on proteins with a common LuxR_C-like conserved domain. The tree is drawn to scale, with branch lengths in the same units as those of the evolutionary distances used to infer the phylogenetic tree. All positions containing alignment gaps and missing data were eliminated only in pairwise sequence comparisons. Bootstrap probabilities (as percentage) were determined from 1000 resamplings. Domain architecture of each group is represented at the side of the tree. The figure is based on the graphical output of the SMART web interface at http://smart.embl-heidelberg.de, with modifications. Sizes and positions of conserved domains are indicated by the labeled symbols. (B) Domain architecture of the EupR cognate histidine kinase. The figure is based on the graphical output of the SMART web interface at http://smart.embl-heidelberg.de, with modifications. Positions of conserved domains are indicated by symbols.

To further characterize EupR, we analyzed in detail its domain composition and its phylogenetic relationship with other proteins showing the same DNA-binding domain. First, both NCBI/CDD and UniProt entries for this protein included an N-terminal signal receiver domain (REC) and a LuxR_C-like DNA-binding helix-turn-helix (HTH) domain. All first 50 hits of the list retrieved after iterative PSI-BLAST, inspected with the CDD domain viewer [27], also showed the same domain composition. Second, we searched *Csal866 *annotation in the specialized Signaling Census database (see Methods), which includes total counts of signal transduction proteins in completely sequenced genomes [28,29]. In this database, *Csal866 *was included as a response regulator of the NarL family. As a matter of fact, published classifications of response regulators based on effector domains refer to NarL-like or NarL/FixJ-like proteins to include response regulators with a REC and a HTH DNA-binding domain, as the first structurally characterized HTH domain was from *E. coli *NarL [14,17]. The DNA-binding C-terminal HTH domain of NarL-like proteins was further proposed as a member of the superfamily of the LuxR_C-like DNA-binding HTH domains [30]. Thus, we made a phylogenetic analysis of EupR and related proteins, all containing the common LuxR_C-like domain. These included well characterized response regulators as well as other homologous but uncharacterized proteins revealed by PSI-BLAST searches, two EupR paralogs present in the *C. salexigens *genome (also classified in the Signaling Census database as response regulators of the NarL family), and "true" LuxR transcriptional regulators related to quorum sensing. All these proteins were aligned by using ClustalW and the phylogenetic tree was constructed using the Neighbor-joining algorithm of the MEGA 4 software. As shown in Figure 8, the vast majority of the proteins were grouped into two subtrees or families. The first subtree comprised two-component response regulators of the NarL/FixJ family, including well characterized proteins such as the *S. meliloti *FixJ regulator (controlling nitrogen fixation genes [31]), the *E. coli *UhpA regulator (controlling the UhpT sugar phosphate transport system [32]), and the *E. coli *NarL protein that controls nitrate- and nitrite-regulated gene expression [33]. All proteins in the first family showed the N-terminal signal receiver phosphoacceptor domain (REC) and the LuxR_C-like domain. Within this family, *C. salexigens *EupR formed a separated branch with other three proteins of unknown function from *Pseudomonas putida*, *Aeromonas salmonicida *and *Vibrio harveyi*. The EupR paralog Csal_2132 (YP 574182) was closely related to the BvgA virulence factors transcription regulator from *Bordetella pertussis *(unpublished), whereas the EupR paralog Csal_3030 (YP 575073) was related to the *S. meliloti *FixJ regulator [31]. The second family included transcriptional regulators that were not response regulators of two components systems, but proteins related to quorum sensing mechanisms. These proteins shared the LuxR_C-like DNA binding domain but showed an N-terminal autoinducer binding domain typical of quorum sensing regulators. Although all these regulators are involved in quorum sensing mediated responses, they control a wide variety of cellular functions, from elastase expression in the case of *P. aeruginosa *LasR [34] to antibiotic production in the case of *P. carotovorum *CarR [35]. The remaining proteins formed separated and independent branches and only showed the LuxR_C-like DNA binding domain. They were involved in different functions like sporulation control as GerE from *B. subtilis *[36] or biofilm formation as PsoR from *P. putida *[37]. All these data indicate that EupR is a response regulator of two-component regulatory systems of the NarL/FixJ family of proteins.

### Identification and analysis of the sensor histidine kinase putatively associated to EupR

The classical two-component regulatory systems require a response regulator protein and a sensor protein, usually a membrane-bound sensor histidine protein kinase [16]. To identify the cognate histidine kinase of EupR, we used the the online application STRING 8.2 (http://string.embl.de/; [38]), a database and web resource dedicated to predict protein-protein interactions including both physical and functional interactions. STRING uses prediction algorithms based on data of neighborhood, gene fusion and co-occurrence across genomes, among others. A total of 21 histidine protein kinases and 29 response regulators are included in the genome of *C. salexigens *(http://www.ncbi.nlm.nih.gov/Complete_Genomes/SignalCensus.html) but only the protein encoded by *Csal869*, located three genes downstream EupR (see Figure 5), was connected with EupR by STRING with a high confidence score (0.772, composed of a neighborhood score of 0.193 and a co-occurrence across genomes score of 0.736). Predictions based on STRING algorithms do not have the specificity of experimental data, but have enough statistical robustness as to be considered reliable [38].

To make a deeper functional *in silico *analysis of this signal transduction protein, we first compared it against several domain databases (see Methods). As Figure 8b shows, we found five distinct domains in the protein: two N-terminal "input" or sensor domains (SSF and PAS-PAC), a transmitter C-terminal region with a His-containing phosphoaceptor HiskA domain and an ATP-binding HATPase domain, and a C-terminal signal receiver domain (REC). The key residues (active site) were conserved in HiskA, HATPase and REC domains. Thus, this protein may be classified as a multi-sensor hybrid histidine kinase. In fact, it was included as such in the Signaling Census database [28,29]. Although sensory domains of histidine kinases are extremely diverse, members of the same family domain typically recognize the same (or very close) substrates [39]. Therefore, we anticipated that the analysis of the two sensory domains in our histidine kinase could help us to predict its putative function. The first one showed homology to transmembrane sensory domains like PutP (Na+/proline symporter-like, in COG database) and SSF (sodium/solute symporter family, in Pfam database). It was preceded by a signal peptide and predicted to form twelve transmembrane helices. The second one, predicted to be cytoplasmatic, showed two PAS subdomains followed by a C-terminal PAC subdomain. In summary, the putative cognate histidine kinase of EupR was predicted to be a hybrid histidine kinase with both transmembrane and cytoplasmic sensor domains, suggesting that it could sense both external and internal conditions, and integrate them. Moreover, our *in silico *analysis supports the hypothesis that it may be the sensor partner of EupR.

## Discussion

In this work, we have characterized the Tn*1732*-induced salt-sensitive mutant CHR95 of *C. salexigens*, which showed a multiple affected phenotype: (i) inability to grow with glucose at high salinity, but not affection in the synthesis of compatible solutes, (ii) a slow growth with glucose at low and optimal salinity, (iii) a reduced uptake and metabolism of glucose, (iv) a deregulated ectoine uptake at any salinity, and specially at low salinity, but unaffected ectoine metabolism, and (v) sensitivity to manganese. This pleiotropic phenotype was due to deletion of three genes by the insertion of Tn*1732*, *acs*, encoding a putative acetyl-CoA synthase, *mntR*, encoding a manganese-dependent transcriptional regulator of the DtxR/MntR family, and *eupR*, encoding a two-component response regulator of the NarL/FixJ family of transcriptional regulators. Transposon Tn*1732 *is a derivative of Tn*1721*, which in turn is a member of the Tn*21 *subgroup of the Tn*3 *family [40]. It has been widely used for generalized insertion mutagenesis in strains of *Halomonas *and *Chromohalobacter*, yielding single mutants [41]. However, as any Tn*1721*-derivative, it may cause deletions and inversions [42]. Thus, deletion of the region comprising *acs*-*eupR*-*mntR *upon Tn*1732 *insertion in CHR95 is not surprising. In fact, in the same mutagenesis experiment in which CHR95 was isolated, we also isolated the salt-sensitive mutant CHR62, showing a deletion of the *ectABC *genes [21,22].

Whereas the sensitivity of strain CHR95 to manganese was correlated with the absence of *mntR*, its inability to grow with glucose at high salt, and the reduced transport and metabolism of glucose at low and optimal salinity (leading to a slow growth with this carbon source) may be related to deletion of the *acs *and/or *eupR *genes. The physiological role of the Acs enzyme is to activate acetate to acetyl-coenzyme A (Ac-CoA), providing the cell with the two-carbon metabolite used in many anabolic and energy generation processes [43]. Besides Acs (YP_572921), *C. salexigens *genome encodes at least one protein (YP_573871) showing a PRK03584 domain of Ac-CoA synthases, and also two more proteins with putative acyl CoA synthase domains (YP_573520 and YP_574569). One or more of these proteins might compensate the lack of Acs in CHR95. In addition, it has been reported that prokaryotic cells have evolved different pathways to obtain Ac-CoA, some of them independent of the *acs *gene [43]. Therefore, with the present data we cannot conclude that deletion of the *acs *gene influenced the ability of strain CHR95 to grow with glucose as the sole carbon source. The role of the response regulator EupR in such a phenotype seems to be more clearly established, as a single *eupR *mutant showed the same growth pattern with glucose as the original mutant CHR95.

Uptake of exogenous compatible solutes is preferred over the synthesis, as it is energetically more favorable to the cells [5]. In *C. salexigens*, the uptake of ectoine, which can be used as a carbon source as well as an osmoprotectant, is maximal at optimal salinity and minimal at low salinity, suggesting that ectoine transport is osmoregulated and most probably devoted to ectoine accumulation from the external medium. In agreement with these transport data, ectoine(s) can be used as carbon source(s) at optimal but not at low salinity [25]. Our previous studies on glucose and ectoine metabolism in this microorganism showed that glucose represses partially ectoine catabolism [25]. However, strain CHR95, which was affected in the transport and metabolism of glucose, did not show an enhanced catabolism of ectoine. These observations indicate that the ability of CHR95 to use ectoine(s) as carbon source at low salinity is decoupled from its impaired glucose catabolism. Rather, it was related to a deregulated ectoine uptake, especially at low salinity. Our results suggest that this phenotype is due to the lack of the two-component response regulator EupR, as a single *eupR *mutant reproduced the ability of CHR95 to use ectoine(s) as carbon source(s) at low salinity. Preliminary data on the expression of a transcriptional fusion between the *C. salexigens teaA *gene, encoding the ectoine binding protein of the TRAP-transporter for ectoine(s), and the *lacZ *reporter gene, revealed that expression of *teaA *in an *eupR *mutant at 0.75 M NaCl is 66% higher than in the wild type (J. Rodriguez-Moya, unpublished results), supporting the hypothesis that EupR is involved in the transcriptional control of ectoine uptake.

In the closely related *H. elongata*, the *teaABC *genes (encoding the osmoregulated TRAP transporter for ectoine) are followed by *teaD*, encoding a putative universal stress protein (USP). Deletion of *teaD *resulted in an enhanced uptake for ectoine by the transporter TeaABC, but it did not affect *teaA *mRNA-levels, excluding a transcriptional regulation mechanism for TeaD. Rather, TeaD was suggested to function either as a translational regulator or as a direct/indirect regulator of TeaABC transport activity [44]. EupR and TeaD proteins do not show homology to each other, as they belong to different protein families and do not share functional domains. Thus, whereas *H. elongata *TeaD shows the conserved sensory domain of cytoplasmic proteins of the Universal stress protein family [44], C. *salexigens *EupR contains a single N-terminal receiver domain and a C-terminal HTH DNA-binding domain of the NarL/FixJ family of response regulators [14,17]. As judged by the fact that the *eupR *mutant is salt-sensitive and grows slower than the wild type with glucose, most probably EupR regulates other processes, besides ectoine uptake, which may or may not be related to the osmostress response. This seems to be also the case of OmpR and MtrA, two response regulators involved in osmoadaptation in *E. coli *[13] and *C. glutamicum *[11], respectively. Our phylogenetic analysis grouped EupR with proteins of unknown functions. Its closest characterized relative was the *E. coli *NarL, which is responsible for the control of nitrate- and nitrite-regulated gene expression [33]. However, assigning protein function based on the function of its closest experimentally characterized homolog is not readily applicable to signal transduction components, as proteins with very similar sequences may have dramatically different biological functions [39]. Therefore, we cannot infer a role of EupR in nitrate- and nitrite-regulated gene expression, besides its involvement in the control of ectoine uptake.

The typical scheme of bacterial two-component signal transduction involves signal sensing by a sensory histidine kinase that leads to its autophosphorylation, followed by phosphoryl transfer to Asp residue in the N-terminal REC domain of the cognate response regulator [16]. However, the cognate response regulator and the histidine kinase are not always encoded in close proximity to each other, which complicates their identification [14]. In any case, presence of a gene in the neighborhood of a response regulator could strengthen the case for the analyzed protein being a histidine kinase [39]. The gene *Csal869*, located three genes downstream of *eupR*, was predicted to be the cognate histidine kinase associated to EupR. This protein satisfies all the key criteria to be considered as the sensory hybrid histidine kinase. The N-terminal sensor domains of the histidine kinases vary greatly in sequence, membrane topology, composition, and domain arrangement. This variability presumably reflects different principles in stimulus perception and processing. For instance, *E. coli *KdpD seems to have a cytoplasmic sensor domain (for K^+^) and also a transmembrane-associated sensing mechanism (osmolality) [15]. The histidine kinase putatively associated to EupR showed two sensor domains. The first one was predicted to form twelve transmembrane helices and was homologous to sodium/solute symporters (SSSF domain). The stimuli sensed by transmembrane sensory domains such as SSF are membrane associated or occur directly within the membrane interface. They include turgor and mechanical stress, ion or electrochemical gradients and transport processes. For instance, the SSF domain is present in *E. coli *PutP [45], which uses the free energy stored in electrochemical Na^+ ^gradients for the uptake of the compatible solute proline. The second sensory domain was predicted to be cytoplasmatic, and showed two PAS subdomains followed by a C-terminal PAC subdomain. Cytoplasmic sensor domains such as PAS detect the presence of cytoplasmic solutes or respond to diffusible or internal stimuli, such as O_2 _or H_2_, or stimuli transmitted by transmembrane sensors.

This redundancy of sensory domains is not rare in nature and in fact a large number of sensor kinases harbor more than one (putative) input domain [15]. The most obvious explanation for the presence of two sensor domains in the protein kinase putatively associated to EupR is that it could sense both external and internal conditions and integrate them. This will be the focus of a further work.

## Conclusions

This work paves the way to the elucidation of the osmosensing and signal transduction pathway leading to the control of ectoine uptake in the model halophilic bacterium *C. salexigens*. Through the characterization of the salt-sensitive mutant CHR95, we found the gene *eupR*, encoding a two-component response regulator of the NarL/FixJ family of transcriptional regulators. In our view, the original annotation of EupR as a "two component LuxR family transcriptional regulator" was imprecise, as the EupR protein is not involved in quorum sensing. However, it was precisely annotated in the specialized Signaling Census database, and further confirmed by our phylogenetic analysis, as a response regulator of the NarL/FixJ family. Our results suggest that EupR is not only involved in the control of ectoine uptake, but also in other processes that might or not be related to the *C. salexigens *osmostress response. Finally, our bioinformatic analysis predicted that the gene *csal869 *encodes a multi sensor hybrid histidine protein kinase which could be the sensory partner of EupR. The presence of two sensor domains in this protein suggest that it could participate in the cross-talk between different signal transduction pathways, as it might be able to sense both external (ions gradient, turgor stress, transport) and internal (cytoplasmatic solutes or proteins, redox state) conditions and integrate them. Future work should focus on (i) elucidating the EupR regulon through transcriptomic analysis, (ii) the *in vivo *analysis of the role of Csal869 as the cognate protein histidine of EupR, and (iii) investigating if the putative EupR histidine kinase could sense the presence of solutes such as ectoine(s) during uptake.

## Methods

### Bacterial strains, plasmids and growth conditions

The bacterial strains and plasmids used in this study are described in Table 3. Strain CHR61, a spontaneous Rf^r ^mutant of *C. salexigens *DSM 3043, was used as the wild type strain. CHR61 displays wild type growth at all conditions tested. *C. salexigens *strains were routinely grown in complex SW-2 medium containing 2% (w/v) total salts *Escherichia coli *was grown aerobically in complex Luria-Bertani (LB) medium M63 [48], which contains 20 mM glucose as the sole carbon source, was used as minimal medium for *C. salexigens*. The osmotic strength of M63 was increased by the addition of a 0.6 to 2.5 M final concentration of NaCl. Although *C. salexigens *can grow in M63 with 0.5 M NaCl, growth is extremely slow at this salinity, and cells take a very long time to reach exponential phase. Therefore, we used M63 with 0.6-0.75 M NaCl as the standard medium for a low salt concentration in all experiments. The pH of all media was adjusted to 7.2 with KOH. Solid media contained 20 g of Bacto agar per liter (Difco). Otherwise stated, cultures were incubated at 37°C in an orbital shaker at 200 rpm. When used, filter-sterilized antibiotics were added at the following final concentrations (μg ml^-1^): ampicillin (Ap), 150 for *E. coli*; chloramphenicol, 25 for *E. coli*; gentamicin (Gm), 20 for *E. coli *and 25 for *C. salexigens*; kanamycin (Km), 50 for *E. coli *and 75 for *C. salexigens*; rifampin (Rf), 25 for *E. coli *and *C. salexigens*; streptomycin (Sm), 20 for *E. coli *and 50 for *C. salexigens *and geneticin (Gn), 20 for for *E. coli *and *C. salexigens*. When used as the sole carbon sources, ectoine and hydroxyectoine (bitop AG, Witten, Germany) were added to the media at a final concentration of 20 mM. Growth was monitored as the optical density of the culture at 600 nm (OD_600_) with a Perkin-Elmer Lambda 25 UV/Vis spectrophotometer.

**Table 3:** Bacterial strains and plasmids used in this study

Strain or plasmid	Relevant genotype and/or description	Source or reference
***C. salexigens *strains**		
DSM 3043^T^	Wild type	[19]
CHR61	Spontaneous Rf^r ^mutant of *C. salexigens *DSM 3043	[21]
CHR95	CHR61 Δ*eupRmntR*::Tn*1732*; Rf^r ^Km^r^	This study
CHR161	CHR61 *mntR*::Ω; Rf^r ^Sm^r ^Spcr	This study
CHR183	CHR61 *eupR*::Ω; Rf^r ^Gn^r^	This study
***E. coli *strain**		
DH5α	*supE44 *Δ(*lac*)*U169 *ϕ80d*lacZ *Δ*M15 hsdR17 recA1 endA1 gyrA96 thi-1 relA1*; host for DNA manipulations	[65]
**Plasmids**		
pKS(-)	Cloning vector; Ap^r^	Stratagene
pHP45Ω	pBR322 derivative carrying the Ω cassette; Ap^r ^Smr Sp^r^	[50]
pHP45Ω*aac*	pBR322 derivative carrying the Ω*aac *cassette; Ap^r ^Gm^r ^Gn^r^	[51]
pRK600	Helper plasmid; Cm^r ^*tra*	[66]
pJQ200-SK	Suicide vector; Gm^r ^*mob sac*	[52]
pSUP102-Gm::Tn*1732*	Mutagenesis plasmid carrying Tn*1732*; Cm^r ^Km^r ^Gmr	[40,49]
pRR1	pKS derivative carrying a 20.8-kb *sac*I fragment from CHR95 including Tn*1732*; Ap^r ^Km^r^	This study
pMntREupR	3-kb *Xba*I-*Apa*I fragment from *C. salexigens *genome (containing *orf1, eupR, mntR*, *orf4*) cloned into pKS; Ap^r^	This study
pHpaIMntr	pMntREupR derivative containing a *Hpa*I recognition site within *mntR*; Ap^r^	This study
pHindIIIEupR	pMntREupR derivative containing a *Hin*dIII recognition site within *eupR*; Ap^r^	This study
PΩMntR	pHpaIMntr derivative with Ω cassette within *mntR*; Ap^r ^Sm^r ^Sp^r^	This study
pΩEupR	pHindIIIEupR derivative with Ω*aac *cassette within *eupR*; Ap^r ^Gn^r ^Gm^r^	This study
pJQMntR	5-kb *Xba*I-*Apa*I fragment from pΩMntR (containing *orf1, eupR, mntR*::Ω, *orf4*) cloned into pJQ200-SK; Gm^r ^Sm^r ^Sp^r^	This study
pJQEupR	5-kb *Xba*I-*Apa*I fragment from pΩEupR (containing *orf1, eupR*::Ω*aac, mntR*, *orf4*) cloned into pJQ200-SK; Gm^r ^Gn^r^	This study

### Conjugal transfer of plasmids

Plasmids were transferred from *E. coli *to *C. salexigens *by triparental mating on SW-2 medium, using pRK600 as a helper plasmid, as described by Vargas et al. [46].

### Methods for nucleic acid manipulation

Plasmid DNA was isolated from *E. coli *with a Wizard Plus SV miniprep kit (Promega), and genomic DNA was isolated with a SpinClean Genomic DNA Purification Kit (Mbiotech). Restriction enzyme digestion and ligation were performed as recommended by the manufacturers (Amersham-Pharmacia Biotech and Fermentas). DNA sequencing was performed by Newbiotechnic (Seville, Spain).

Transposon mutagenesis was performed by conjugal transfer of pSUP102-Gm::Tn*1732 *from *E. coli *SM10 [40,49] to *C. salexigens *strain CHR61. Matings were carried out by mixing the donor and recipient cultures at a ratio of 1:4 (100 μl of donor, 400 μl of recipient). The mixed cultures were washed with sterile SW-2 medium to eliminate the antibiotics. The pellet was resuspended in 100 μl of SW-2 and placed on a 0.45-μm pore filter on SW-2 solid media (which allows the growth of *E. coli *and the putative salt-sensitive mutants of *C. salexigens*). After overnight incubation at 30°C, cells were resuspended in 20% (v/v) sterile glycerol and, after appropriate dilutions, inoculated on SW-2 + rifampicin + Km plates at a density resulting in about 100-200 colonies per plate. Colonies from these master plates were transferred with sterile toothpicks to duplicate M63 plates, one contained 2.7 M NaCl and the other contained 0.5 M NaCl. Plates were incubated at 37°C and inspected for colonies that had grown at 0.5 M but not at 2.7 M NaCl. One of these colonies was selected for further experiments and was named CHR95.

To clone the DNA region flanking the Tn*1732 *insertion in CHR95, genomic DNA of this mutant was digested with *Sac*I, ligated to *Sac*I-digested pKS(-) and the ligation mix was used to transform *E. coli *DH5α cells. From Km^r ^Ap^r ^colonies, the plasmid pRR1, containing the transposon Tn*1732 *within one *Sac*I fragment of about 20.7-kb, was isolated.

To generate *C. salexigens *mutants affected in *mntR *or *eupR*, a 3.054-bp fragment from genome containing 680 bp of *orf1*, *eupR*, *mntR *and *orf4 *was PCR amplified with *Pfu *Turbo DNA polymerase (Stratagene) by using two synthetic oligonucleotides (MntRfw: 5'-CATGCTGATC**T**AGACGCTGTCGG-3' and MntRrv: 5'-GCAGGCG**G**GC**C**CCATCTG-3') that were modified (residues in bold) to introduce a *Xba*I and an *Apa*I site, respectively (underlined). The resulting PCR fragment was digested with *Xba*I and *Apa*I, and the 3,054-bp fragment generated was cloned into pKS bluescript to give plasmid pMntREupR. Subsequently, an *Hpa*I or *Hin*dIII recognition site was introduced in *mntR *or *eupR *respectively, using the PCR-based QuikChange Site-Directed Mutagenesis Kit (Stratagene) and the following oligonucleotides: MntRHpa_fw: 5' CCGAATTGGTCGAGGACTATGT**TA**A**C**GAGATTGCGCATTTGC-3', MntRHpa_rv: 5'-GCAAATGCGCAATCTC**G**T**TA**ACATAGTCCTCGACCAATTCGG-3', EupRHind_fw: 5'-GCACGGCGCACCACCGGCG**AAG**CTTCGCTTCCCCAGATGACC-3', and EupRHind_rv: 5'- GGTCATCTCGGGAAGCGAAG**CTT**CGCCGGTGGTGCGCCGTGC-3', that were modified (residues in bold) to introduce the corresponding restriction sites. The resultant plasmids, pHpaIMntR and pHindIIIEupR were linearized with the enzyme *Hpa*I or *Hin*dIII and ligated to 2-kb *Sma*I or *Hin*dIII fragments from pHP45-Ω [50] or pHP45-Ωaac [51], containing the Ω interposons for insertional mutagenesis (Sm^r ^or Gn^r^). The resulting plasmids were named pΩMntR and pΩEupR. To recombine the *mntR or eupR *mutations into the *C. salexigens *chromosome, 5-kb *Xba*I-*Apa*II fragments from pΩMntR or pΩEupR were cloned into the suicide vector pJQSK200 (Gm^r^) [52] to give plasmids pJQMntR and pJQEupR, which were mobilized into the *C. salexigens *wild type strain by triparental mating. Mutant strains resulting from a double homologous recombination event were identified as Sm^r ^Gm^s^, or Gn^r ^Gm^s ^colonies on SW-2 plates containing 10% sucrose. Two of these colonies were purified for further analysis and were named CHR161 (*mntR*::Ω) and CHR183 (*eupR*::Ω*aac*). Insertions of the omega cassette in CHR161 and CHR183 were confirmed by PCR and sequencing.

### Determination of sensitivity to Mn

To determine the sensitivity of *C. salexigens *strains to Mn, we used fresh plates of a modified SW-2 medium containing less than 1 mM of SO_4_Mg (to avoid interference of Mg^2+ ^with Mn^2+^), which was additioned with 0.5 to 2.5 mM MnCl_2_. An overnight culture of each strain (100 μl) was spread onto the assay plate and growth was observed after incubation at 37°C for 48 h.

### Determination of ectoine uptake

Cells grown overnight in SW-2 were subcultured at a 1:100 dilution in glucose M63 medium containing 0.75, 1.5 or 2.5 M of NaCl, and grown up to exponential phase (OD_600 _ca. 0.5). Transport was initiated by adding [^14^C]-ectoine to 0.2 ml of bacterial suspensions and incubating the cultures at room temperature. The [^14^C]-ectoine (5.5 MBq mM) was prepared biologically from *Brevibacterium linens *as described [53] and was added at a final concentration of 87 μM. During 2 min, 50 μl of samples were taken at 30-s intervals, and transport was terminated by rapid filtration through Whatman GF/F discs (Fisher Bioblock, Illkirch, France). The cells were quickly washed twice with 2 ml of isotonic M63 medium. The filters were solubilized in scintillation fluid and radioactivity was determined in a Packard Liquid Scintillation Analyzer, 1600 TR (Perkin Elmer, Courtabeouf, France). Transport rates were expressed as nmol min-1 OD-1 unit.

### Determination of the metabolic fate of [^14^C]-glucose

Cells grown overnight in SW-2 were subcultured at a 1:100 dilution in M63 containing 1.5 M NaCl and 20 mM glucose, and grown up to exponential phase (OD_600 _*ca*. 0.5). 2 ml samples were centrifuged, resuspended in 1.5 M NaCl M63 to an OD_600 _of *ca*. 0.6 and transferred to a Warburg flask. ^14^C-labelled glucose (5.5 mCi/mmol, 390000 dpm/5 μl) was added at a final concentration of 100 μM to the samples. After different incubation times at 37°C, 1 ml of sample was centrifuged for 10 min at 16000 g; 50 μl of supernatant was taken (twice) and radioactivity was measured as above, indicating the glucose remaining in the supernatant (S, dpm ml-^1^). Cell pellet was resuspended in 20 μl of H_2_O, extracted with 80 μl of pure ethanol and centrifuged for 10 min at 13000 rev min-^1^. The ethanolic supernatant was dried in a Speed Vac (Savant Instruments, Holbrook, NY, USA), and the solid residue was resuspended in 50 μl of H_2_O. An aliquot of 10 μl was used to measure the radioactivity caused by the ethanol-soluble 28 compounds synthesized from glucose (ESF, dpm per OD unit). The ethanol insoluble pellet was resuspended in 50 μl of H_2_O and used to measure the radioactivity caused by the ethanol-insoluble compounds synthesized from glucose (EIF, dpm per OD unit).

### Determination of the metabolic fate of [^14^C]-ectoine

Cells grown overnight in SW-2 were subcultured at a 1:100 dilution in M63 containing 1.5 M NaCl and 20 mM glucose and grown up to exponential phase (OD_600 _*ca*. 0.5). Two independent 2 ml samples were centrifuged, resuspended in 1.5 M NaCl M63 to an OD_600 _of *ca*. 0.6 and transferred to a Warburg flask. ^14^C-labelled ectoine (5.5 MBq mM) was added at a final concentration of 87 μM to the samples. Glucose was added to one of the samples at a final concentration of 20 mM. After 2-h incubation at 37°C, the fate of radioactive ectoine was analysed as follows: (i) respired radioactive CO_2 _was trapped on a strip of 3 MM Whatman filter paper moistened with 50 μl of 6 mol l-^1 ^of KOH and ^14^CO_2 _production (dpm per OD_600 _unit) was measured by liquid scintillation; (ii) 1 ml of sample was centrifuged for 10 min at 16000 g; 50 μl of supernatant was taken (twice) and radioactivity was measured as above, indicating the ectoine remaining in the supernatant (S, dpm ml-^1^); and (iii) cell pellet was resuspended in 20 *μ*l of H_2_O, extracted with 80 μl of pure ethanol and centrifuged for 10 min at 13 000 rev min-^1^. The ethanolic supernatant was dried in a Speed Vac (Savant Instruments, Holbrook, NY, USA), and the solid residue was resuspended in 50 μl of H_2_O. An aliquot of 10 μl was used to measure the radioactivity caused by the ethanol-soluble compounds synthesized from ectoine (ESF, dpm per OD unit). The ethanol insoluble pellet was resuspended in 50 μl of H_2_O and used to measure the radioactivity caused by the ethanol-insoluble compounds synthesized from ectoine (EIF, dpm per OD unit).

### Phylogenetic analysis

Phylogenetic and molecular evolutionary analyses were conducted using MEGA version 4 [54]. *C. salexigens *EupR and other LuxR family proteins including well characterized members of different subclasses with a common LuxR-C-like conserved domain and others different domains were included in the phylogenetic analyses. We also included some uncharacterized proteins with a high similarity to *C. salexigens *EupR, including two paralogs present in *C. salexigens *genome.

The sequences were aligned with clustalW (1.6) using a BLOSUM62 matrix and manually edited. The phylogenetic tree was inferred using the Neighbor-joining method [55] and the evolutionary distances were computed using the Poisson correction method. The rate variation among sites was modelled with a gamma distribution (shape parameter = 1.5) and all the positions containing gaps and missing data were eliminated only in pairwise sequence comparisons. The robustness of the tree branches was assessed by performing bootstrap analysis of the Neighbor-joining data based on 1000 resamplings [56].

### DNA and protein sequences analysis

The sequence of the *C. salexigens *genome is available at NCBI microbial genome database (http://www.ncbi.nlm.nih.gov/genomes/lproks.cgi Ac N°: NC_007963). Sequence data were analyzed using PSI-BLAST at NCBI server http://www.ncbi.nlm.nih.gov/BLAST. Promoter sequences were predicted using BGDP Neural Network Promoter Prediction http://www.fruitfly.org/seq_tools/promoter.html. Signal peptides and topology of proteins were predicted using SMART 6 (http://smart.embl-heidelberg.de/; [57,58]). Other programs and databases used in proteins topology and functional analysis were STRING 8.2 (http://string.embl.de/; [38]) KEGG (http://www.genome.ad.jp/kegg/pathway/ko/ko02020.html; [59]), Signaling census (http://www.ncbi.nlm.nih.gov/Complete_Genomes/SignalCensus.html; [28,29]), PROSITE (http://www.expasy.org/prosite/; [60]), BLOCKS (http://blocks.fhcrc.org/; [61]), Pfam (http://pfam.janelia.org/; [62]), CDD (http://www.ncbi.nlm.nih.gov/Structure/cdd/cdd.shtml; [27]), InterProScan (http://www.ebi.ac.uk/interpro/; [63]), and Phobius (http://www.ebi.ac.uk/Tools/phobius/; [64]).

## Authors' contributions

JRM and MA performed the majority of the experiments, participated in bioinformatics analysis, study design, and in crafting of the manuscript. MRB, MJ, and FIG performed some growth experiments and RMN analyses. JJN and CV conceived the study, participated in the design, coordination, bioinformatic analysis, and crafting of the manuscript. All authors have read and approved the final manuscript.
